# Do Elderly Patients With Stage I–II Hepatocellular Carcinoma Benefit From More Radical Surgeries? A Population-Based Analysis

**DOI:** 10.3389/fonc.2020.00479

**Published:** 2020-04-16

**Authors:** Qiu-Qiang Zhang, Pan-Yi-Sha Wu, Mugahed ALBahde, Lu-Fei Zhang, Zhu-Ha Zhou, Hua Liu, Yu-Feng Li, Wei-Lin Wang

**Affiliations:** ^1^Department of Hepatobiliary and Pancreatic Surgery, The Second Affiliated Hospital, Zhejiang University School of Medicine, Hangzhou, China; ^2^Clinical Medicine Innovation Center of Precision Diagnosis and Treatment for Hepatobiliary and Pancreatic Disease of Zhejiang University, Hangzhou, China; ^3^Department of General Surgery, School of Medicine, Sir Run Run Shaw Hospital, Zhejiang University, Hangzhou, China; ^4^Department of Hepatobiliary and Pancreatic Surgery, The First Affiliated Hospital, Zhejiang University School of Medicine, Hangzhou, China; ^5^Key Laboratory of Precision Diagnosis and Treatment for Hepatobiliary and Pancreatic Tumor of Zhejiang Province, Hangzhou, China; ^6^Research Center of Diagnosis and Treatment Technology for Hepatocellular Carcinoma of Zhejiang Province, Hangzhou, China; ^7^Clinical Research Center of Hepatobiliary and Pancreatic Diseases of Zhejiang Province, Hangzhou, China

**Keywords:** SEER, early stage, surgery, geriatric, survival

## Abstract

**Background and Aims:** The best treatment modalities for elderly patients with stage I–II HCC (hepatocellular carcinoma) remain controversial in an era of a shortage of liver donors.

**Methods:** From the SEER database (Surveillance, Epidemiology, and End Results program), 2,371 elderly patients were sampled as Cohort 1. OS (Overall Survival) and CSS (Cancer-Specific Survival) were compared between the Non-surgery and Surgery groups. A stratification analysis in a CSS Cox model was also conducted among sub-groups, and propensity score matching was performed to generate Cohort 2 (746 pairs), reducing the influences of confounders.

**Results:** For Cohort 1, the median follow-up times of the Non-surgery and Surgery groups were 11 months (95% CI, confidence interval: 9.74–12.26) vs. 49 months (44.80–53.21) in OS, and 14 months (12.33–15.67) vs. 74 months (64.74–83.26) in CSS, respectively. In the stratification analysis, for the elderly patients (age >= 70 years), Larger Resection was associated with a higher HR (hazard ratio) than Segmental Resection: 0.30 (95% CI, confidence interval: 0.22–0.41) vs. 0.29 (0.21–0.38) in 70–74 year-olds; 0.26 (0.18–0.38) vs. 0.23 (0.16–0.32) in 75–79 year-olds; 0.32 (0.21–0.49) vs. 0.21 (0.13–0.32) in those 80+ years old. For Cohort 2, a similar result could be seen in the CSS Cox forest plot. The HRs of Larger Resection and Segmental Resection were 0.27 (0.21–0.33) and 0.25 (0.20–0.31), respectively.

**Conclusions:** It is cautiously recommended that, when liver transplantation is not available, segmental or wedge liver resection is the better treatment choice for elderly patients with stage I–II HCC (AJCC edition 6), especially those over 70 years old, compared with other surgeries, based on the SEER data.

## Introduction

Hepatocellular carcinoma (HCC) is considered worldwidely to be one of the most malignant tumors (1). Today, with the increasingly aging global population, the proportion of elderly patients (age >= 65 years) with HCC is also becoming higher and higher each year (2). Elderly patients with HCC usually have worse prognostic survivals than younger patients do due to their poor health status or comorbidities, such as COPD (chronic obstructive pulmonary disease), cerebral stroke, and organ dysfunctions (3). It is therefore very challenging to find the best treatments of geriatric patients with HCC. And there are really some very special and distinctive characteristics in nursing and surgeries, especially for those diagnosed with stage I–II HCC (AJCC edition 6). On the one hand, there are many therapy options for them (4), including surgeries (e.g., segmental liver resection, hemihepatectomy, liver transplantation), radiation, and chemotherapy; on the other hand, controversy remains over which kinds of treatments are better for them, offering longer survival and less invasiveness, in an era of liver donor shortage (5). It is also undetermined whether these elderly patients benefit substantially more from the more radical surgical therapies, e.g., hemihepatectomy or liver transplantation, compared with the less invasive surgeries, such as radio-frequency ablation or segmental liver resection. For the present, there are few studies that investigate this issue with convincing and dependable huge-scale data such as ours (6, 7).

As of now, the large Surveillance, Epidemiology, and End Results (SEER) database in the USA, which covers almost 28% of the US population (8), is an ideal and perfect data pool for oncologic studies worldwide (9, 10). In this study, we sampled the elderly patients (age >= 65 years, stage I–II HCC) from the SEER database to try to determine whether more radical surgeries should be suggested for them and to explore the highest-impact and key factors for survival, particularly when liver transplantation is not available.

## Patients and Methods

### Data Source

The SEER database (version 2019) is a public, free clinical records platform (11, 12) that comprises demographic and oncologic information of cancer patients from 18 registries across the USA, renewed online every year. In this research, we used SEER-stat software (version 8.3.5) to obtain the clinical data of those patients who were age >= 65 years, diagnosed as stage I–II HCC (AJCC edition 6), and hospitalized between January 1, 2004, and December 31, 2011. The overall regime of our research design is shown in the flowchart in Supplementary Figure 1.

### Study Cohort

A total of 2,371 patients were finally sampled as Cohort 1. Since some variables, such as the specific surgery, tumor size, grade of morphology, and age, are highly involved in the prognostic outcome of HCC treatments, we excluded patients without complete data on these variables. Besides, follow-up months, overall vital status, and the cause-specific death variable were vital for the OS (overall survival) and CSS (cancer-specific survival) analysis, so we also ruled out patients without complete ascertainment and definite information on these parameters. Patients who did not have HCC diagnosis as their only or first of more than one tumors were also excluded. In order to reduce the influence of the confounding factors further, we used the PSM (propensity score matching) method to mimic randomized controlled trials (RCT) by producing a more balanced new dataset, Cohort 2, with 1,492 patients (746 pairs) in all. The PSM was based on logistic regression analysis of the variables in Cohort 1, which covered nearly all items except the grouping variable, Specific Surgery.

### Demographic and Clinical Data Pretreatment

The patients were divided broadly into Non-surgery and Surgery groups. Some layers of the variables from the original data were merged. For example, the Larger Resection group was the integration of the lobectomy and the extended lobectomy in SEER. Furtherly, we regrouped the patients by Specific Surgery, which was composed of None, Local Destruction (local tumor destruction, e.g., Radio-Frequency Ablation), Segmental Resection (covering wedge resection), Larger Resection (including lobectomy and extended lobectomy), and Liver Transplantation. Although there is very limited information about the Radiation and Chemotherapy of the patients in SEER, we attempted to cover these variables by transforming them into binaries with two levels (No and Yes). AFP and Fibrosis score data were also collected in our study.

### Statistical Analysis

The baseline demographic data for Non-surgery and Surgery groups were compared through the Student's *t*-test or χ2 test. Patients still alive at December 31, 2011, were censored in the OS analysis, while, in the CSS analysis, those who died from other disease causes except HCC were also censored. The accumulated OS and CSS probabilities were plotted, respectively, and the differences between the two groups were compared by the Kaplan-Meier method with a log-rank test. Cox proportional hazards regression analyses were performed with both a univariate model and a multivariate model in Cohort 1. Besides, the stratification analyses of Age, Grade of morphology, Tumor size, and HCC Stage were wholly conducted in a cross-table by Specific Surgery (including five sub-groups). In addition, a forest plot of hazard ratios was made from the multivariate CSS Cox analysis in Cohort 2.

All statistical tests were evaluated by the significance criterion *P* < 0.05 (two-sided), and the hazard ratios with 95% confidence interval (CI) are also shown in the study. All statistical analyses were conducted using SPSS 23.0 for Windows (SPSS, Chicago, IL, USA) and R software (version × 64 3.5.3). The Institutional Ethics Committee of The Second Affiliated Hospital of Zhejiang University School of Medicine considered the study exempt.

## Results

In our study, 2,371 patients with an affirmative diagnosis of stage I–II HCC and older than 64 years old were selected from the SEER database as Cohort 1. Among these cases, there were 1,283 patients who received surgical treatments, including Local Destruction (LD), Segmental Resection (SR), Larger Resection (LR), Liver Transplantation (LT), while 1,088 cases did not. The baseline demographic characteristics are shown in Table 1. Those who received surgical treatments were a little younger than the Non-surgery patients, 72.1(±5.73) years old vs. 75.1(±6.84) years old, *P* < 0.001. There were no statistical differences (*P* > 0.05) between the Non-surgery and Surgery groups in some variables, namely Gender, Year at diagnosis, and Stage (AJCC edition 6), while the statistical differences can be seen in the other variables. The median follow-up times of the Non-surgery and Surgery groups were 11 months (95% CI: 9.74–12.26) vs. 49 months (44.80–53.21) in OS, and 14 months (12.33–15.67) vs. 74 months (64.74–83.26) in CSS, respectively. As shown in both OS (*P* < 0.0001) and CSS (*P* < 0.0001) plots (Supplementary Figure 2), the Surgery group had a higher survival curve than the Non-surgery group. In the CSS survival Cox proportional hazard ratio models of both univariate analysis (UVA) and multivariate analysis (MVA), it was demonstrated that the survival probabilities were robustly associated with certain factors, e.g., Surgery overall (MVA: HR, hazard ratio, 0.76; 95% CI: 0.76–0.76; *P* < 0.001), Tumor size (>= 1 cm and <3 cm) (MVA: HR, 1.66; 95% CI: 1.44–1.92; *P* < 0.001), and Poor differentiation (MVA: HR, 1.57; 95% CI: 1.34–1.83; *P* < 0.001) (Table 2). Intriguingly, the variable Age was statistically correlated with worse CSS survival only in the univariate Cox model (UVA: HR, 1.04; 95% CI: 1.03–1.05; *P* < 0.001), while the Stage (AJCC edition 6) factor became associated with worse CSS survival only in the multivariate Cox model (MVA: HR, 1.25; 95% CI: 1.11–1.40; *P* < 0.001). Further, in the stratification analyses (Age, Tumor size, Grade of Morphology, and Stage) of the univariate CSS Cox model (Table 3), it was shown that Liver Transplantation had the best survival in nearly all ages bands: HR 0.13 (CI: 0.09–0.20) in 65–69, HR 0.06 (CI: 0.03–0.15) in 70–75, HR 0.11 (CI: 0.02–0.78) in 80+. Meanwhile, Larger Resection had better survival than Segmental Resection only in the 65–69 age band, HR 0.33 (CI: 0.24–0.44) vs. HR 0.38 (CI: 0.29–0.50). However, unexpectedly, at Age >= 70, Larger Resection did not show better survival than Segmental Resection. Similar results could be noticed in some other variables, e.g., Tumor size >= 1 cm and Stage II (Table 3).

**Table 1:** Baseline characteristics.

**Terms**	**No. of Patients (%)**		***P*-value**
	**Non-surgery(*N* = 1088)**	**Surgery(*N* = 1283)**	
**Age (years)**			<0.001
mean (SD)	75.1 (6.84)	72.1 (5.73)	
**Age group**			<0.001
65-69 years	276 (25.4)	520 (40.5)	
70-74 years	273 (25.1)	366 (28.5)	
75-79 years	232 (21.3)	239 (18.6)	
>= 80 years	307 (28.1)	158 (12.3)	
**Gender**			0.827
Female	348 (32.0)	405 (31.6)	
Male	740 (68.0)	878 (68.4)	
**Year at diagnosis**			0.351
2004–2006	315 (29.0)	401 (31.3)	
2007–2009	439 (40.3)	484 (37.7)	
2010–2011	334 (30.7)	398 (31.0)	
**Race**			<0.001
White	759 (69.8)	822 (64.1)	
Black	107 (9.8)	93 (7.2)	
A.I./A.N.^*^	14 (1.3)	10 (0.8)	
Asian / P.I.^Δ^	207 (19.0)	356 (27.7)	
Unknown	1 (0.1)	2 (0.2)	
**Marital status**			<0.001
Unmarried	447 (41.1)	403 (31.4)	
Married	607 (55.8)	849 (66.2)	
Unknown	34 (3.1)	31 (2.4)	
**Stage (AJCC 6th)**			0.427
I	761 (69.9)	878 (68.4)	
II	327 (30.1)	405 (31.6)	
**Grade of morphology**			<0.001
Well	501 (46.0)	416 (32.4)	
Moderately	415 (38.1)	644 (50.2)	
Poorly	160 (14.7)	197 (15.4)	
Undifferentiated	12 (1.1)	26 (2.0)	
**Tumor size**			<0.001
<1 cm	864 (79.4)	1166 (90.9)	
>=1 and <3 cm	212 (19.5)	114 (8.9)	
>=3 cm	12 (1.1)	3 (0.2)	
**Radiation**			<0.001
None	1017 (93.5)	1257 (98.0)	
Yes	71 (6.5)	26 (2.0)	
**Chemotherapy**			<0.001
None	636 (58.5)	1031 (80.4)	
Yes	452 (41.5)	252 (19.6)	
**AFP**			0.002
Negative	273 (25.1)	410 (32.0)	
Borderline	2 (0.2)	2 (0.2)	
Positive	534 (49.1)	554 (43.2)	
Unknown	279 (25.6)	317 (24.7)	
**Fibrosis score**			<0.001
0-4	52 (4.8)	175 (13.6)	
5–6	147 (13.5)	229 (17.8)	
Unknown	889 (81.7)	879 (68.5)	

* *American Indian/Alaska Native*;

Δ *Asian/Pacific Islander*.

**Table 2:** Univariate and multivariate analysis of CSS Cox model.

**Terms**	**Univariate Cox analysis**			**Multivariate Cox analysis**		
	**HR**	**(95% CI)**	***P*-value**	**HR**	**(95% CI)**	***P*-value**
**Age**	1.04	(1.03–1.05)	<0.001	1.02	(0.99–1.05)	0.129
**Age group**						
65–69 years	1.00			1.00		
70–74 years	1.17	(1.02–1.34)	0.026	0.91	(0.75–1.11)	0.345
75–79 years	1.57	(1.36–1.82)	<0.001	1.02	(0.75–1.39)	0.895
>=80 years	1.89	(1.63–2.18)	<0.001	0.89	(0.55–1.44)	0.645
**Gender**						
Female	1.00			1.00		
Male	0.90	(0.81–1.00)	0.055	0.96	(0.85–1.07)	0.447
**Year of diagnosis**						
2004–2006	1.00			1.00		
2007–2009	0.95	(0.84–1.07)	0.382	0.90	(0.80–1.03)	0.117
2010–2011	0.85	(0.75–0.98)	0.021	0.81	(0.71–0.93)	0.003
**Race**						
White	1.00			1.00		
Black	1.15	(0.96–1.38)	0.139	1.03	(0.85–1.24)	0.761
A.I./A.N.^∮^	0.89	(0.52–1.54)	0.680	0.87	(0.50–1.52)	0.635
Asian/P.I.^§^	0.67	(0.59–0.77)	<0.001	0.67	(0.59–0.76)	<0.001
unknown	1.47	(0.37–5.90)	0.585	1.91	(0.47–7.71)	0.363
**Marital status**						
Unmarried	1.00			1.00		
Married	0.82	(0.74–0.91)	0.000	0.93	(0.83–1.04)	0.211
Unknown	0.78	(0.56–1.09)	0.153	0.83	(0.60–1.17)	0.293
**Stage (AJCC 6th)**						
I	1.00			1.00		
II	1.06	(0.95–1.18)	0.295	1.25	(1.11–1.40)	0.000
**Grade of morphology**						
Well	1.00			1.00		
Moderately	0.90	(0.81–1.01)	0.080	1.14	(1.01–1.28)	0.032
Poorly	1.31	(1.13–1.52)	0.001	1.57	(1.34–1.83)	<0.001
Undifferentiated	1.10	(0.73–1.66)	0.644	1.39	(0.92–2.11)	0.121
**Tumor size**						
<1 cm	1.00			1.00		
>=1 and <3 cm	1.97	(1.72–2.26)	<0.001	1.66	(1.44–1.92)	<0.001
>=3 cm	1.76	(0.97–3.18)	0.063	1.23	(0.68–2.24)	0.499
**Specific surgery**						
None	1.00			1.00		
LD*	0.44	(0.38–0.51)	<0.001	0.58	(0.58–0.58)	<0.001
SR**	0.28	(0.24–0.33)	<0.001	0.34	(0.34–0.34)	<0.001
LR***	0.29	(0.25–0.35)	<0.001	0.32	(0.32–0.32)	<0.001
LT^Δ^	0.09	(0.07–0.13)	<0.001	0.12	(0.12–0.12)	<0.001
Surgery overall	0.30	(0.27–0.33)	<0.001	0.76	(0.76–0.76)	<0.001
**Radiation**						
None	1.00			1.00		
Yes	1.31	(1.03–1.66)	0.030	0.87	(0.68–1.11)	0.275
**Chemotherapy**						
None	1.00			1.00		
Yes	1.09	(0.97–1.21)	0.134	0.72	(0.64–0.81)	<0.001
**AFP**						
Negative	1.00			1.00		
Borderline	1.06	(0.34–3.30)	0.923	1.39	(0.44–4.35)	0.576
Positive	1.54	(1.36–1.75)	<0.001	1.37	(1.20–1.57)	<0.001
Unknown	1.48	(1.29–1.71)	<0.001	1.39	(1.20–1.61)	<0.001
**Fibrosis score**						
1–4	1.00			1.00		
5–6	1.41	(1.12-1.76)	0.003	1.27	(1.00-1.61)	0.046
Unknown	1.71	(1.41-2.07)	<0.001	1.17	(0.96-1.43)	0.12

Univariate and multivariate analysis of CSS Cox proportional hazard ratio analysis before propensity score matching. CSS, cancer-specific survival; A.I./A.N.^∮^ American Indian/Alaska Native; Asian/P.I.^§^ Asian/Pacific Islander; LD*, Local Destruction; SR**, Segmental Resection; LR***, Larger Resection, LT^Δ^, Liver Transplantation.

**Table 3:** Stratification analysis.

**Strata**	**Non-surgery**	**Local destruction**	**Segmental resection**	**Larger resection**	**Liver transplantation**
	***N*/reference**	***N*/HR (95% CI)**	***N*/HR (95% CI)**	***N*/HR (95% CI)**	***N*/HR (95% CI)**
**Age group**					
65–69 years	276	127	135	126	132
	1	0.51 (0.40–0.67)	0.3 8 (0.29–0.50)	0.33 (0.24–0.44)	0.13 (0.09–0.20)
70–74 years	273	105	127	97	37
	1	0.49 (0.37–0.65)	0.29 (0.21–0.38)	0.30 (0.22–0.41)	0.06 (0.03–0.15)
75–79 years	232	91	81	64	3
	1	0.37 (0.27–0.49)	0.23 (0.16–0.32)	0.26 (0.18–0.38)	0.11 (0.02–0.78)
>=80 years	307	58	52	47	1
	1	0.41 (0.28–0.58)	0.21 (0.13–0.32)	0.32 (0.21–0.49)	–
**Tumor size**					
<1 cm	864	366	369	258	173
	1	0.48 (0.41–0.56)	0.30 (0.26–0.36)	0.28 (0.23–0.35)	0.10 (0.07–0.14)
>=1 and <3 cm	212	15	25	74	0
	1	0.41 (0.22–0.77)	0.25 (0.15–0.43)	0.29 (0.21–0.41)	–
>=3 cm	12	0	1	2	0
	1	–	0.01 (0.00–17.77)	0.02 (0.00–5.17)	–
**Grade of morphology**					
Well	501	176	103	74	64
	1	0.42 (0.34–0.52)	0.25 (0.18–0.34)	0.23 (0.16–0.34)	0.09 (0.05–0.16)
Moderately	415	163	212	183	86
	1	0.46 (0.37–0.58)	0.27 (0.21–0.34)	0.28 (0.22–0.36)	0.09 (0.05–0.14)
Poorly	160	40	67	67	23
	1	0.45 (0.30–0.68)	0.30 (0.21–0.43)	0.29 (0.20–0.42)	0.41 (0.28–0.61)
Undifferentiated	12	2	13	10	1
	1	0.08 (0.08–0.69)	0.11 (0.03–0.35)	0.14 (0.04–0.48)	–
**Stage (AJCC 6th)**					
I	761	273	294	220	91
	1	0.40 (0.33–0.48)	0.25 (0.21–0.30)	0.28 (0.23–0.35)	0.09 (0.06–0.14)
II	327	108	101	114	82
	1	0.58 (0.44–0.74)	0.39 (0.29–0.52)	0.31 (0.23–0.41)	0.09 (0.06–0.15)

*Cancer-specific survival comparison stratified by age group, tumor size, grade of morphology, and AJCC stage before PSM. PSM, propensity score matching; HR, hazard ratio; CI, confidence interval*.

**Figure 1:**
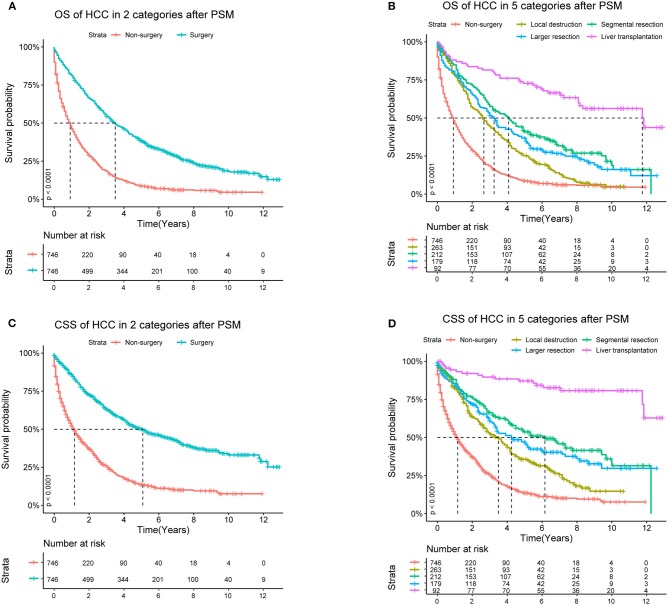
OS (Overall Survival) and CSS (Cancer Specific Survival) analyses of HCC (Hepatocellular Carcinoma) patients in Cohort 2 after PSM (Propensity Score Matching).

**Figure 2:**
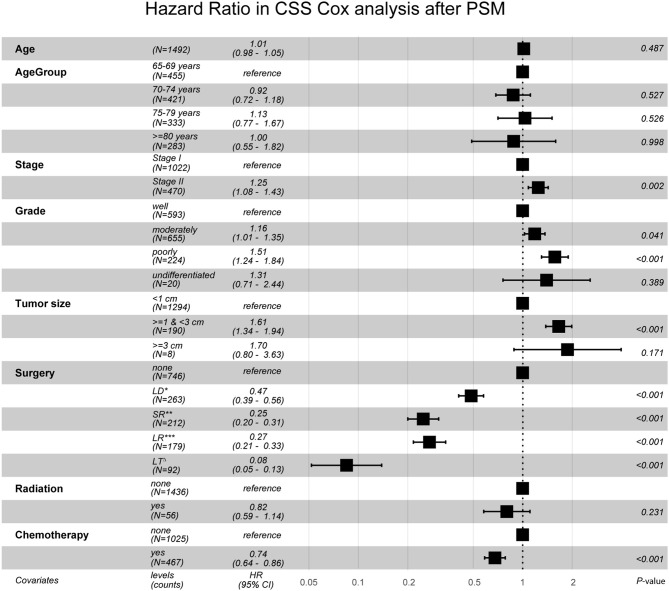
Hazard Ratio in CSS Cox analysis after PSM. CSS, cancer specific survival; PSM, propensity score matching; LD*, Local Destruction; SR**, Segmental Resection; LR***, Larger Resection, LT^Δ^, Liver Transplantation. Global events: 968; Global *p*-value (Log-Rank): 2.8355e-90.

After PSM, in Cohort 2 with 1,492 cases (746 pairs), the distributions became more balanced in nearly all of the variables (Supplementary Table 1), and more detailed survival plots were completed on OS and CSS. As depicted, the Non-surgery group had a lower survival curve than the Surgery group did, and Liver Transplantation had the highest survival curve (*P* < 0.0001) (Figure 1). It is also shown that Larger Resection had a better survival curve than Local Destruction but a worse one than Segmental Resection and Liver Transplantation, both in the OS (*P* < 0.0001) and CSS (*P* < 0.0001). The results of the multivariate CSS Cox analysis of Cohort 2 are clearly demonstrated in the forest plot in Figure 2. In accordance with the stratification results of Cohort 1, Liver Transplantation was the best protective factor (*P* < 0.001), and Larger Resection did not show a better survival (*P* < 0.001) than did Segmental Resection.

## Discussion

Given the increasing population of elderly people worldwide, geriatric patients constitute a large proportion of HCC patients each year, but only a few studies have focused on the optimization of surgical treatments for them (13, 14). In our study, all of the patients who received surgical treatments, including Local Destruction, Segmental Resection, Larger Resection, and Liver Transplantation, had better survival than did the Non-surgery patients, which is consistent with the previous studies (2, 10, 12). Among the Surgery groups, Liver Transplantation was the best treatment on the condition that there were enough liver donors. Local Destruction, such as via RFA (radio-frequency ablation) or PEI (percutaneous ethanol injection), was implicated as having a worse survival rate than other surgeries, which was also reported previously (4, 15, 16). However, unexpectedly, the Larger Resection sub-group (extended liver resection, e.g., hemihepatectomy or lobectomy) was not associated with better survival than Segmental Resection (including wedge resection, usually less than a hemihepatectomy or lobectomy). Meanwhile, some authors (17) might think that RFA has good indications for HCC patients with tumor size <2 cm and no vascular invasion, but this is not true in our study. We found that Local Destruction had higher HRs than did Segmental Resection, at both Stage I (level 1) and Stage II (level 2). Accordingly, in all age groups and tumor size groups, Local Destruction had higher HRs than did surgery groups (Table 3). Local Destruction, such as via RFA, may enable good control of local foci in the near future, but it is usually associated with higher recurrence rates and higher death rates in long-term follow-up (18). Therefore, if liver transplantation was not available, for the elderly patients with stage I–II HCC, wedge or segmental liver resection would be better choices for longer survival associated with relatively less invasiveness and a faster post-operational recovery.

In order to interrogate the significant factors that impact the survival of elderly patients with Stage I–II HCC, the precise selection of the patients is an important prerequisite, such as Age >= 65 years old, with confirmative clinical diagnosis of HCC. Those without HCC as the only primary cancer or as the first of more than one tumors and those without complete clinical data (e.g., follow-up time, morphological information) were both ruled out to make our study more convincing and rigorous. In addition, the cut-off line of the research interval was also very meaningful. Since the latest SEER data available online are always lagging behind by about 3 years and the present SEER coding system began in 2004, we chose the period 2004–2011 so that each patient in our study had the potential to help to calculate the 5 year survival rate. All of these methods were taken to ensure a more balanced and reasonable dataset, although the sample size of patients became smaller than in previous studies (2, 19).

In fact, we have collected as many variables as possible in Table 2, based on the available data from the SEER database. We have not screened out any variables from the UVA to MVA model, since all the possible factors and their overall effects should be considered in the end, which is much more necessary and dependable in reality. We found that Surgery, Tumor size, and Poor differentiation of morphology are the impactive factors for the post-operation survival in both the UVA and MVA of Cohort 1. Age is an influential factor in survival in UVA, but the effects are not obvious in MVA, which may be due to the sample size no being large enough. Stage is one of the key factors in MVA, and emphasis should be placed upon it in overall analyses (20).

Our study is clinical-value-oriented, and it becomes much more scientifically persuasive after stratification and PSM, based on the giant SEER database (21). Age, Tumor size, Grade of morphology, and AJCC Stage often have vital impacts on HCC patients' survival, so it was deemed necessary to perform a stratification analysis with the original data of Cohort 1. This revealed that segmental or wedge liver resection is generally a better choice for elderly patients above 69 years with Stage I–II HCC when liver transplantation is not available. This is somewhat different from some other research findings (4, 16, 22, 23). After PSM, in Cohort 2, there were no statistical differences (*P* > 0.05) between the Non-surgery and the Surgery groups in nearly all variables, so the effects of different surgeries could be more clearly compared. As shown in the forest plot, for those elderly people with stage I–II HCC, Larger Resection (extended surgery) does not show better survival than Segmental Resection does, which is consistent with the results of Cohort 1. As far as we know, this point has been reported for the first time in our study with the giant SEER dataset. It may provide some help to surgeons when they are confronted with the dilemma of how to make the best choice for geriatric patients with early-stage HCC with a poor health status when liver transplantation is unavailable.

However, there are also some drawbacks that need to be noticed. In fact, there is a lot of information that is unavailable in SEER but is closely correlated with post-operative survival, such as the details of the surgery, laboratory results, post-operational radiation, and systemic chemotherapy, as well as the performance status and severity of dysfunction of the liver or other organs (7, 16, 20, 24). Although the fibrosis score is provided, it is not sufficient to estimate the severity of cirrhosis. Also, staging information is only for the TNM system (American Joint Committee on Cancer), and there is not enough data to enable staging with the BCLC (Barcelona Clinical Liver Cancer) stage system, in which Child-Pough classification and performance status are considered (19). Besides, there also is no information about comorbidities, such as chronic obstructive pulmonary disease (COPD), coronary artery disease, and renal dysfunction (3), which are often seen in elderly patients. The specifics of chemotherapy, such as whether TACE (transarterial embolization) (25) or preoperative adjuvant chemotherapy (5) were used, is not available in SEER either. In addition, although we performed PSM to decrease the impacts of confounders and to abolish the selection bias to some degree, there are still some flaws in PSM itself (12), e.g., the smaller scale and the undermined representativeness after PSM, so the conclusions should be taken prudently. We may, in future, perhaps be able to draw on the Medicare Billing database (5, 13) for more comprehensive clinical data to make a more objective, reasonable, and convincing study of geriatric patients.

## Conclusion

In summary, it is cautiously recommended that surgeries for elderly patients with stage I–II HCC have much better survival outcomes than non-surgical choices do, based on the limited data of SEER. Wedge or segmental liver resection has better survival than do the other surgeries (including local tumor destruction, extended liver resection, etc.) when liver transplantation is not available. More prospective randomized controlled clinical trials with a larger number of patients may be needed for further validation.

## Data Availability Statement

The raw data supporting the conclusions of this article is free and publicly accessible in SEER database.

## Ethics Statement

The studies involving human participants were reviewed and approved by The Institutional Ethnics Committee of The Second Affiliated Hospital of Zhejiang University School of Medicine. The patients/participants provided their written informed consent to participate in this study.

## Author Contributions

W-LW has access to all of the data in this study and takes responsibility for the integrity and accuracy of the data analyses. Q-QZ and W-LW: study concept and design. Q-QZ: drafting of the manuscript. Q-QZ and Z-HZ: statistical analysis. W-LW: study supervision. All authors: critical revision of the manuscript for important intellectual content, acquisition, analysis, or interpretation of data.

### Conflict of Interest

The authors declare that the research was conducted in the absence of any commercial or financial relationships that could be construed as a potential conflict of interest.

## References

[1] Torre LA Bray F Siegel RL Ferlay J Lortet-Tieulent J Jemal A. Global cancer statistics, 2012. CA Cancer J Clin. (2015) 65:87–108. 10.3322/caac.21262 25651787

[2] Oweira H Petrausch U Helbling D Schmidt J Mannhart M Mehrabi A. Early stage hepatocellular carcinoma in the elderly: a SEER database analysis. J Geriatr Oncol. (2017) 8:277–83. 10.1016/j.jgo.2017.03.002 28389117

[3] Peters NA Javed AA He J Wolfgang CL Weiss MJ. Association of socioeconomics, surgical therapy, and survival of early stage hepatocellular carcinoma. J Surg Res. (2017) 210:253–60. 10.1016/j.jss.2016.11.042 28457336

[4] Ciccarese F Caturelli E Felder M Farinati F Frigo AC Morisco F. Survival benefit of liver resection for patients with hepatocellular carcinoma across different barcelona clinic liver cancer stages: a multicentre study. J Hepatol. (2014) 62:617–24. 10.1016/j.jhep.2014.10.037 25450706

[5] Shaya FT Breunig IM Seal B Mullins CD Chirikov V V Hanna N. Comparative and cost effectiveness of treatment modalities for hepatocellular carcinoma in SEER-medicare. Pharmacoeconomics. (2014) 32:63–74. 10.1007/s40273-013-0109-7 24293197

[6] Hirokawa F Hayashi M Miyamoto Y Asakuma M Shimizu T Komeda K. Surgical outcomes and clinical characteristics of elderly patients undergoing curative hepatectomy for hepatocellular carcinoma. J Gastrointest Surg. (2013) 17:1929–37. 10.1007/s11605-013-2324-0 24002762

[7] Yu B Ding Y Liao X Wang C Wang B Chen X. Radiofrequency ablation versus surgical resection in elderly patients with early-stage hepatocellular carcinoma in the era of organ shortage. Saudi J Gastroenterol. (2018) 24:317–25. 10.4103/sjg.SJG_261_18 30117492 PMC6253917

[8] Zhang G Li R Zhao X Meng S Ye J Zhao L. Validation of the American Joint Committee on Cancer eighth edition staging system in patients undergoing hepatectomy for hepatocellular carcinoma: a US population-based study. J Surg Res. (2018) 222:55–68. 10.1016/j.jss.2017.09.044 29273376

[9] Abdel-Rahman O. Assessment of the discriminating value of the 8th AJCC stage grouping for hepatocellular carcinoma. HPB. (2018) 20:41–8. 10.1016/j.hpb.2017.08.017 28882455

[10] Kamarajah SK. Fibrosis score impacts survival following resection for hepatocellular carcinoma (HCC): a Surveillance, end results and epidemiology (SEER) database analysis. Asian J Surg. (2018) 41:551–61. 10.1016/j.asjsur.2018.01.001 29454570

[11] Fu J Ruan H Zheng H Cai C Zhou S Wang Q. Impact of old age on resectable colorectal cancer outcomes. PeerJ. (2019) 7:e6350. 10.7717/peerj.6350 30792941 PMC6378948

[12] Jiang Y-Q Wang Z-X Deng Y-N Yang Y Wang G-Y Chen G-H. Efficacy of Hepatic resection vs. Radiofrequency ablation for patients With very-early-stage or early-stage hepatocellular carcinoma: a population-based study with stratification by age and tumor size. Front Oncol. (2019) 9:113. 10.3389/fonc.2019.00113 30863723 PMC6400103

[13] Parikh ND Marshall VD Green M Lawrence TS Razumilava N Owen D. Effectiveness and cost of radiofrequency ablation and stereotactic body radiotherapy for treatment of early-stage hepatocellular carcinoma: an analysis of SEER-medicare. J Med Imaging Radiat Oncol. (2018) 62:673–81. 10.1111/1754-9485.12754 29877615

[14] Han B Yao H Shao L Guo X Han L Romeiro FG. Selection of treatment modalities for hepatocellular carcinoma at stages T1 and T2: a preliminary analysis based on the surveillance, epidemiology, and end results registry database. J Buon. (2018) 23:611–21. 30003727

[15] Xu J Noda C Erickson A Mokkarala M Charalel R Ramaswamy R. Radiofrequency ablation vs. cryoablation for localized hepatocellular carcinoma: a propensity-matched population study. Anticancer Res. (2018) 38:6381–6. 10.21873/anticanres.12997 30396961

[16] Kim GA Shim JH Kim MJ Kim SY Won HJ Shin YM. Radiofrequency ablation as an alternative to hepatic resection for single small hepatocellular carcinomas. Br J Surg. (2016) 103:126–35. 10.1002/bjs.9960 26572697

[17] Jianyong L Lunan Y Dajiang L Wentao W. Comparison of open liver resection and RFA for the treatment of solitary 3-5-cmhepatocellular carcinoma: a retrospective study. BMC Surg. (2019) 19:195. 10.1186/s12893-019-0663-9 31842844 PMC6916101

[18] Xu XL Liu X Di Liang M Luo BM. Radiofrequency ablation versus hepatic resection for small hepatocellular carcinoma: systematic review of randomized controlled trials with meta-analysis and trial sequential analysis. Radiology. (2018) 287:461–72. 10.1148/radiol.2017162756 29135366

[19] Zhao J Mao J Li W. Association of tumor grade with long-term survival in patients with hepatocellular carcinoma after liver transplantation. Transplant Proc. (2019) 51:813–9. 10.1016/j.transproceed.2018.12.033 30979469

[20] Sposito C Battiston C Facciorusso A Mazzola M Muscarà C Scotti M Propensity score analysis of outcomes following laparoscopic or open liver resection for hepatocellular carcinoma. Br J Surg. (2016) 103:871–80. 10.1002/bjs.10137 27029597

[21] Zhang N Gu J Yin L Wu J Du MY Ding K. Incorporation of alpha-fetoprotein(AFP) into subclassification of BCLC C stage hepatocellular carcinoma according to a 5-year survival analysis based on the SEER database. Oncotarget. (2016) 7:81389–401. 10.18632/oncotarget.13232 27835609 PMC5348400

[22] Lei JY Zhong JJ Yan LN Zhu JQ Wang WT Zeng Y. Response to transarterial chemoembolization as a selection criterion for resection of hepatocellular carcinomas. Br J Surg. (2016) 103:881–90. 10.1002/bjs.9864 27027978

[23] Su M Zhao Y Liu J. The role of definitive local treatment in metastatic hepatocellular carcinoma patients: a SEER-based study. Medicine. (2018) 97:e0020. 10.1097/MD.0000000000010020 29517658 PMC5882428

[24] Malhotra J Mhango G Gomez JE Smith C Galsky MD Strauss GM. Adjuvant chemotherapy for elderly patients with stage I non-small-cell lung cancer ≥4 cm in size: an SEER-Medicare analysis. Ann Oncol. (2015) 26:768–73. 10.1093/annonc/mdv008 25600562

[25] Mironov O Jaberi A Kachura JR. Thermal ablation versus surgical resection for the treatment of stage T1 hepatocellular carcinoma in the surveillance, epidemiology, and end results database population. J Vasc Interv Radiol. (2017) 28:325–33. 10.1016/j.jvir.2016.11.001 28073607

