# Supplementary material for: Do Elderly Patients With Stage I–II Hepatocellular Carcinoma Benefit From More Radical Surgeries? A Population-Based Analysis
Source: Front Oncol. 2020 Apr 16;10:479. doi: 10.3389/fonc.2020.00479 (PMC7176977; doi:10.3389/fonc.2020.00479)
Supplement: Supplementary file 1 [file Table_1.docx]

**Supplementary Table 1**

Demographics of HCC patients after PSM (*N*=1,492)

Terms

**No. of Patients (%)**

***P*-value**

| **Non-Surgery (***N***=746) Surgery (***N***= 746)** | | | |
| --- | --- | --- | --- |
| **Age (years)** |  |  | 0.764 |
| mean (SD) | 73.7 (6.36) | 73.6 (6.05) |  |
| **Age group** |  |  | 0.834 |
| 65-69 years | 235 (31.5) | 220 (29.5) |  |
| 70-74 years | 206 (27.6) | 215 (28.8) |  |
| 75-79 years | 167 (22.4) | 166 (22.3) |  |
| >= 80 years | 138 (18.5) | 145 (19.4) |  |
| **Gender** |  |  | 0.468 |
| female | 228 (30.6) | 241 (32.3) |  |
| male | 518 (69.4) | 505 (67.7) |  |
| **Year at diagnosis** |  |  | 0.965 |
| 2004-2006 | 233 (31.2) | 231 (31.0) |  |
| 2007-2009 | 288 (38.6) | 293 (39.3) |  |
| 2010-2011 | 225 (30.2) | 222 (29.8) |  |
| **Race** |  |  | 0.779 |
| White | 502 (67.3) | 506 (67.8) |  |
| Black | 78 (10.5) | 68 (9.1) |  |
| American Indian/Alaska Native | 8 (1.1) | 6 (0.8) |  |
| Asian/Pacific Islander | 158 (21.2) | 165 (22.1) |  |
| unknown | 0 (0.0) | 1 (0.1) |  |
| **Marital status**  unmarried | 280 (37.5) | 273 (36.6) | 0.926 |
| married | 446 (59.8) | 452 (60.6) |  |
| unknown | 20 (2.7) | 21 (2.8) |  |
| **Stage (AJCC 6th edition)** |  |  | 0.824 |
| I | 513 (68.8) | 509 (68.2) |  |
| II | 233 (31.2) | 237 (31.8) |  |
| **Grade of morphology** |  |  | 0.856 |
| well | 304 (40.8) | 289 (38.7) |  |
| moderately | 323 (43.3) | 332 (44.5) |  |
| poorly | 110 (14.7) | 114 (15.3) |  |
| undifferentiated | 9 (1.2) | 11 (1.5) |  |
| **Tumor size** |  |  | 0.354 |
| <1 cm | 648 (86.9) | 646 (86.6) |  |
| >=1 & <3 cm | 92 (12.3) | 98 (13.1) |  |
| >=3 cm | 6 (0.8) | 2 (0.3) |  |
| **Specifics surgery** |  |  | - |
| none | 746 (100) | - |  |
| Local Destruction | - | 263 (35.3) |  |
| Segmental Resection | - | 212 (28.4) |  |
| Larger Resection | - | 179 (24.0) |  |
| Liver Transplantation | - | 92 (12.3) |  |
| Surgery overall | - | 746 (100) |  |

**Radiation** none

yes

Chemotherapy

| none | 508 (68.1) | 517 (69.3) |
| --- | --- | --- |
| yes | 238 (31.9) | 229 (30.7) |

715 (95.8) 721 (96.6)

31 (4.2) 25 (3.4)

0.414

0.615

AFP

negative borderline

positive unknown

Fibrosis score

0-4

5-6

214 (28.7) 212 (28.4)

1 (0.1) 2 (0.3)

343 (46.0) 348 (46.6)

188 (25.2) 184 (24.7)

44 (5.9) 43 (5.8)

119 (16.0) 120 (16.1)

0.966

0.992

unknown 583 (78.2) 583 (78.2)
