# Supplementary figures and images for: Do Elderly Patients With Stage I–II Hepatocellular Carcinoma Benefit From More Radical Surgeries? A Population-Based Analysis
Source: Front Oncol. 2020 Apr 16;10:479. doi: 10.3389/fonc.2020.00479 (PMC7176977; doi:10.3389/fonc.2020.00479)

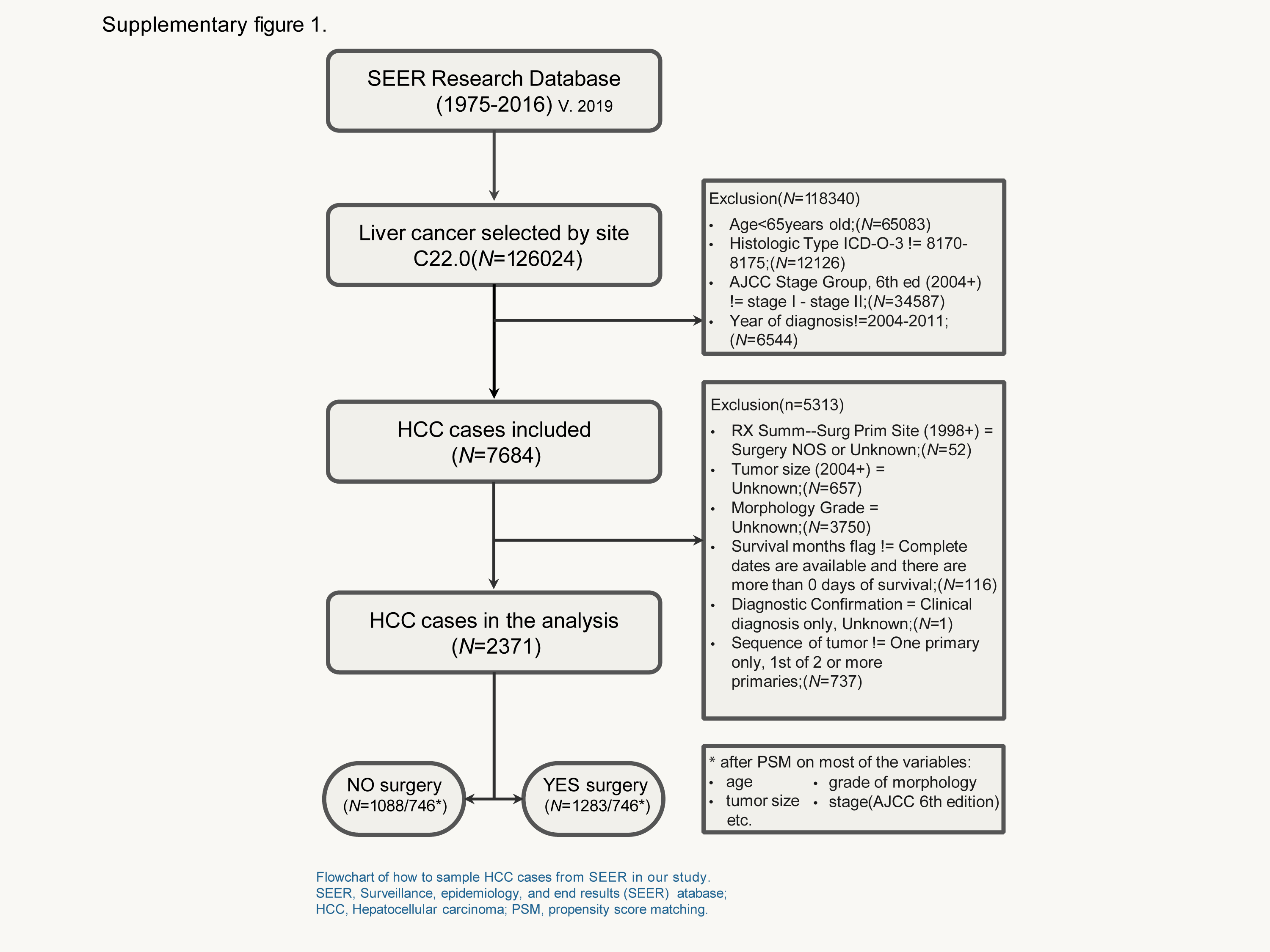

Supplement: Supplementary file 2 [file Image_1.TIF]

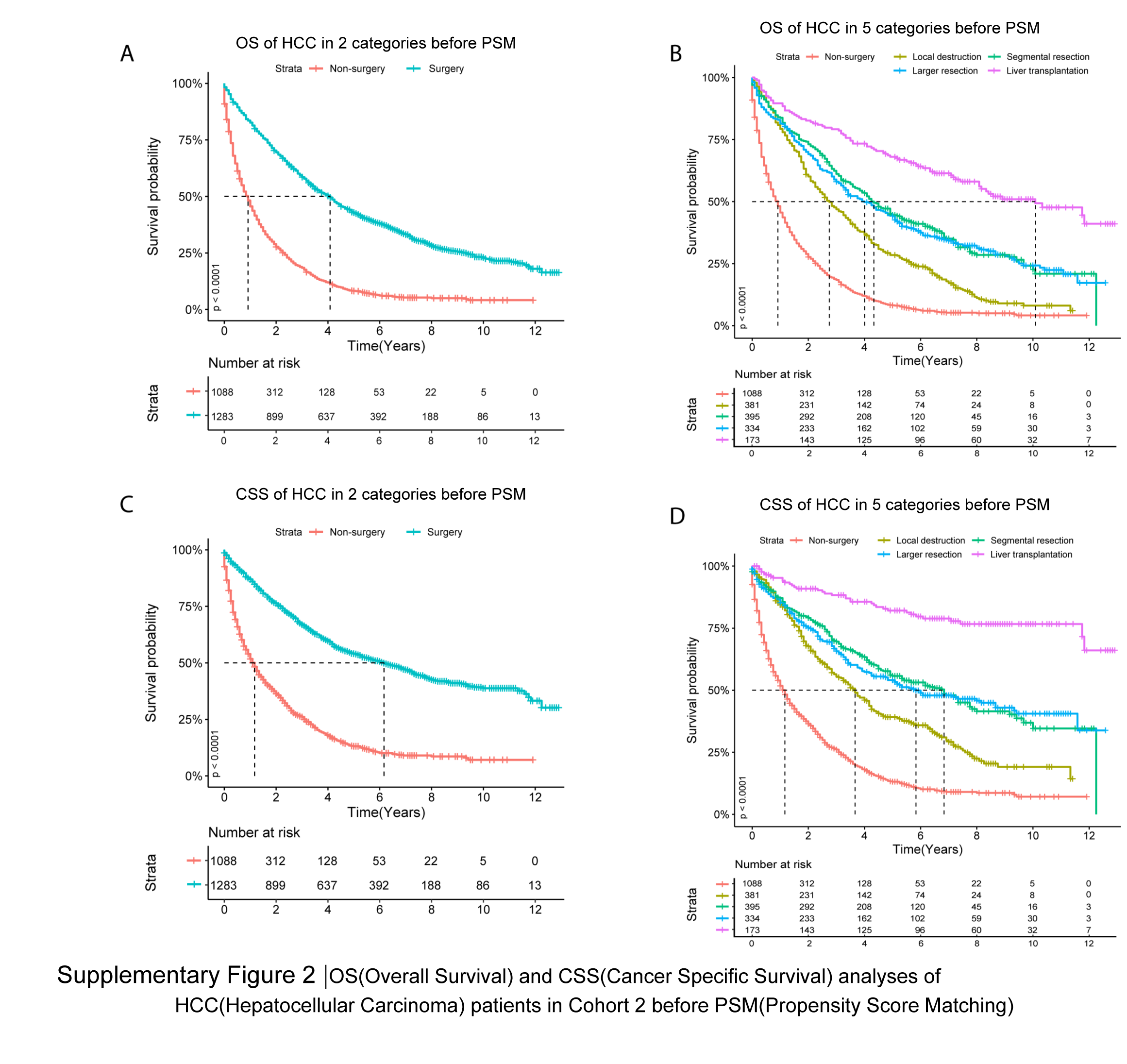

Supplement: Supplementary file 3 [file Image_2.tif]
